# Nonantibiotic Treatment of Clinical Multidrug‐Resistant Bacterial Isolates Using Chitosan Nanoparticles as a Carrier for the Supernatant of Mesenchymal Stem Cells

**DOI:** 10.1155/cjid/3465372

**Published:** 2026-05-28

**Authors:** Sareh Bagheri-Josheghani, Mahmood Saffari, Hamed Mirzaei, Zahra Sadat Fateminasab, Mozhgan Derakhshan-Sefidi, Somaye Rashki, Bita Bakhshi

**Affiliations:** ^1^ Infectious Diseases Research Center, Kashan University of Medical Sciences, Kashan, Iran, kaums.ac.ir; ^2^ Department of Microbiology and Immunology, Faculty of Medicine, Kashan University of Medical Sciences, Kashan, Iran, kaums.ac.ir; ^3^ Research Center for Biochemistry and Nutrition in Metabolic Diseases, Institute for Basic Sciences, Kashan University of Medical Sciences, Kashan, Iran, kaums.ac.ir; ^4^ Department of Microbiology, Saveh University of Medical Sciences, Saveh, Iran; ^5^ Student Research Committee, Kashan University of Medical Sciences, Kashan, Iran, kaums.ac.ir; ^6^ Department of Laboratory Sciences, Sirjan School of Medical Sciences, Sirjan, Iran; ^7^ Department of Medical Bacteriology, Faculty of Medical Sciences, Tarbiat Modares University, Tehran, Iran, modares.ac.ir

**Keywords:** antibacterial, bacterial infections, drug resistance, nanotechnology, stem cells

## Abstract

This study aimed to evaluate the antimicrobial potential of the supernatant (conditioned medium) of mesenchymal stem cells–derived conditioned medium (MSCs CM), incorporated in chitosan nanoparticles (CS NPs), against multidrug‐resistant (MDR) clinical isolates. Dynamic light scattering (DLS), zeta potential (ZP), and scanning electron microscopy (SEM) assays were used to characterize CS NPs. MSCs CM was incorporated into CS NPs, and its release pattern from the composite (MSCs CM–CS NPs) was evaluated. Antibacterial properties of MSCs CM–CS NPs, as well as MSCs CM and CS NPs, were assessed by the microbroth dilution technique. The average particle size of most CS NPs was 85.2 nm, and their zeta potential was 32.1 mV. SEM findings supported the morphology of the prepared CS NPs and the synthesized composite (MSCs CM–CS NPs). Entrapment efficiency of the new construct was 71%. The antimicrobial activity of the new construct (MSCs CM–CS NPs: 1000 μg/mL + 0.05%) against MDR bacteria was significantly higher compared with MSCs CM (1000 μg/mL) and CS NPs (0.05%) each alone. This composite exhibited the synergistic antibacterial activity of the combination of MSCs CM and CS NPs, resulting in a more potent dose‐dependent antibacterial activity against MDR clinical isolates.

## 1. Introduction

Multidrug‐resistant(MDR) bacteria are the cause of various clinical problems worldwide, especially in developing countries. Various factors are involved in the emergence of MDR bacteria, including the use and abuse of antibiotics in diverse settings such as animal husbandry, aquaculture, and community; the use of several agents with broad‐spectrum antimicrobial activities; and the absence of continuous antibacterial surveillance in healthcare settings. Globalization of commercial food, migratory birds, and international travel are other factors contributing to the global spread of MDR bacteria [1]. Biofilms reduce the effectiveness of antibacterial drugs in biological extracellular matrices; they also help MDR organisms escape host immune responses. Currently, the major focus in the field of drug resistance is on how to reduce the population of MDR organisms by antibacterial and antibiofilm agents [2].

So far, many antimicrobial agents have been used to treat human infectious diseases in clinical settings. Unfortunately, the widespread usage of some antibacterial agents has led to their reduced efficacy and withdrawal. However, to date, the pharmaceutical industry has been able to identify very few alternative antimicrobial compounds for those that have lost their efficacy in the treatment of most infectious diseases. Today, drug resistance is one of the greatest public health challenges, which creates many problems for both patients and physicians. Therefore, there is an urgent need for more extensive efforts to develop and explore new antimicrobial drugs to increase effective therapeutic options against resistant species [3]. Recently, nanodelivery systems have emerged as a novel, promising method for targeted drug delivery, which improves the administration and efficacy of pharmaceutical compounds and overcomes the limitations of traditional therapeutic methods. Jabir et al. examined the antitumor effects of CALNN and TNF peptides encapsulated in gold nanoparticles (NPs) as a carrier for drug delivery. This novel drug delivery system displayed high cytotoxicity against cancer cells by inducing apoptosis [4]. Recent innovations in drug delivery systems have attracted much attention as potential novel methods for treating infections caused by antibiotic‐resistant pathogens. In a study, the effectiveness of L‐glutaminase as an antimicrobial agent obtained from clinical isolates of *Lactobacillus gasseri* BRLHM was investigated. The purified L‐glutaminase showed substantial antimicrobial effects against MDR *Pseudomonas aeruginosa* isolates [5]. The antibacterial activity of chitosan and its derivatives against Gram‐positive and Gram‐negative pathogens has been investigated in various studies [6]. In a study, the biomedical application of chitosan/polycaprolactone nanofibers loaded with *Nigella sativa* extract was investigated. The results showed that PCL/CS/NS nanofibers were effective in treating and preventing bacterial wound infections [7]. Rad et al. proposed a nanochitosan that exhibited antimicrobial effects against *Listeria monocytogenes* [8].

In another study, chitosan nanoparticles (CS NPs) showed antimicrobial activity against cariogenic streptococci in vitro; this nanochitosan (5 mg/mL) also exhibited an inhibitory effect against *Streptococcus mutans* biofilm formation [9]. In another study by Bahroudi et al., the conditioned medium (CM) derived from mesenchymal stem cells–derived conditioned medium (MSCs CM) exhibited strong dose‐dependent antibacterial activity against *Vibrio cholerae* biofilm formation [10].

Saberpour et al. investigated the antibiofilm and antibacterial properties of MSCs CM coupled with CS NPs (MSCs CM–CS NPs) against MDR *V*. *cholerae* isolates. Their study results revealed a higher antibacterial activity of the composite compared with the controls, which was due to the synergistic effect of its components. Furthermore, MSCs CM and MSCs CM–CS NPs seemed to be more efficient against MDR isolates than CS NPs alone, and the controls [11].

Human bone marrow‐derived MSCs (BM‐MSCs) secrete factors that synergistically interact with antibiotics and indirectly enhance their antibacterial activity and facilitate neutrophil activation [12].

Previous studies have shown that the use of MSCs CM–CS NPs is a new treatment method that could be employed to inhibit the growth of bacterial pathogens. No study has so far evaluated the antimicrobial activities of MSCs CM–CS NPs against MDR pathogens [11]. The synergic activity of the combination of MSCs‐CM with CS NPs as a new therapeutic approach efficiently inhibits bacterial growth. The present research aimed to investigate the in vitro antibacterial activities of CS NPs as a delivery tool for MSCs‐CM (supernatant) against MDR clinical isolates without fear of developing antimicrobial resistance.

## 2. Materials and Methods

### 2.1. Bacterial Strains

*P*. *aeruginosa* (ATCC 27853), *Klebsiella pneumoniae* (NCTC 5056), *Enterobacter cloacae* (PTCC 1003), *Staphylococcus aureus* (PTCC 1337), and *Escherichia coli* (ATCC 25922) were used in this study. Antibiotic susceptibility of the isolates (*Pseudomonas aeruginosa*, *Klebsiella pneumoniae*, *Enterobacter cloacae*, *and Escherichia coli*) to the antibiotics imipenem, meropenem, piperacillin, ampicillin, ampicillin–sulbactam, piperacillin–tazobactam, ceftazidime, cefepime, cefotaxime, ceftriaxone, amikacin, gentamicin, levofloxacin, ciprofloxacin, tetracycline, and trimethoprim–sulfamethoxazole (MAST, Merseyside, UK), and antibiotic susceptibility of *Staphylococcus aureus* to ampicillin, oxacillin, cefoxitin, imipenem, meropenem, erythromycin, clindamycin, vancomycin, tetracycline, rifampin, and trimethoprim–sulfamethoxazole (cotrimoxazole) were determined using the Kirby–Bauer disk diffusion method on Mueller–Hinton agar (Merck, Germany).

Multidrug resistance was described as showing resistance to at least one agent in ≥ 3 CLSI‐recommended antibiotic categories (Clinical and Laboratory Standards Institute, United States, 2018) [13]. These bacterial isolates used in this study were clinical isolates obtained from hospital sources.

### 2.2. Isolation and Characterization of Human BM‐MSCs

Caco‐2 cell line (IBRC10094) (IBRC, Iran, Tehran) and human BM‐MSCs (IBRC10094) (IBRC, Iran, Tehran) were prepared from the Iranian Biological Resource Center. BM‐MSCs were confirmed in our previous research via assessing their differentiation potential into adipocytes and osteoblasts by the immunohistochemistry (IHC) technique. The expression of CD34, CD45, CD44, and CD73 surface antigens as MSCs‐specific biomarkers was assessed via the flow cytometry method [10].

The CM from BM‐MSCs was collected as follows: 1 × 10^6^ BM‐MSCs were plated in a 75 cm^2^ tissue culture flask and then serum‐free DMEM (Sigma‐Aldrich) was added. When the BM‐MSCs reached approximately 90% confluency, the cells were cultured in serum‐free DMEM for 72 h. The CM from BM‐MSCs at Passage 4 was concentrated by centrifugation at 4000 rpm for 30 min at 4°C. After centrifugation, the supernatant was collected and sterilized using 0.22 μm filters. The concentrated CM was aliquoted into microtubes and stored at −80°C until further use [11].

### 2.3. Synthesis of NPs

First, chitosan (degree of deacetylation 75%–85%; molecular weight 50,000–190,000 Da, based on viscosity) obtained from (Sigma‐Aldrich, USA) was used without further purification and added to a 1% acetic acid solution.

Then, sodium tripolyphosphate (STPP, 0.1% w/v) (Sigma‐Aldrich, USA) was added to phosphate‐buffered saline solution (PBS) 1:3 (v/v). Finally, the TPP solution was mixed with the chitosan solution (2:5) while stirring at room temperature [14]. By employing the dynamic light scattering (DLS) (Zetasizer Nano ZS, Malvern, Worcestershire, UK) method, the zeta potential and size distribution of the prepared CS NPs were determined. The particles’ shape was also determined using scanning electron microscopy (SEM) (FESEM, Tescan Mira3) method.

### 2.4. Preparation of MSCs CM‐Loaded NPs

To prepare the MSCs CM–CS NPs composite, MSCs CM was incorporated into CS NPs as reported previously [11].

At all stages of this research, MSCs CM (1000 μg/mL), CS NPs (0.05%), and MSCs CM–CS NPs (1000 μg/mL + 0.05%) were used with prespecified concentrations.

The shape of the particles was estimated by the SEM technique. Also, zeta potential and particle size distribution were determined by the DLS method.

### 2.5. Determination of Entrapment Efficiency (EE)

The bicinchoninic acid (BCA) assay technique was used for protein quantification in the solution. The following formula was used to calculate the EE:

%EE = [(Drug added ‐ Free “unentrapped drug”)/Drug added] × 100 [15].

In this study: EE = (Total amount of protein in MSCs CM‐loaded protein in MSCs CM–CS NPs/Total amount of protein in MSCs CM) × 100.

### 2.6. Evaluation of the Release Assay

The protein release pattern was measured in vitro at two different pH values (3.2 and 7.2) at 37°C after 72 h by the BCA assay kit.

The protein release rate was adjusted as reported previously [11]. PBS was employed as a blank sample. Each experiment was performed twice.

### 2.7. In Vitro Antibacterial Potential by Microbroth Dilution Assay

To assess the antimicrobial activity of the new construct and its constituents (CS NPs and MSCs supernatant) against MDR clinical isolates, a broth microdilution assay was used with slight modifications as previously detailed [11]. In brief, serial dilutions were prepared with 100 μL of MSCs CM–CS NPs (1000 μg/mL +0.05%), MSCs CM (1000 μg/mL), and CS NPs (0.05%) in LB broth‐containing 96‐well microplates and serially diluted (1:2–1:64).

Then, bacterial suspensions (5 × 10^5^ colony forming unit [CFU]/mL) and Mueller–Hinton broth (0.1 mL) (MHB) were added to the 96‐well microplates.

The plates were then incubated at 37°C for 24 h, and the optical density (OD) of the 96‐well plates was read at 620 nm after the incubation time.

Wells containing bacterial suspensions without antimicrobials, as well as Caco‐2 cell supernatant (100 μL) rather than MSCs supernatant, were regarded as positive and negative controls, respectively. PBS was regarded as a negative control (free of bacteria and compounds) to confirm the absence of bacterial growth and contamination. Control wells containing (Caco‐2 cell supernatant and bacterial suspensions) were used as a positive control. Since Caco‐2 cells lack known antimicrobial activity, unlike some MSCs, their supernatant supported bacterial growth and served as a positive control. This provided a baseline against which any growth inhibition observed in test wells could be attributed specifically to the added other compounds.

### 2.8. MTT Assay

Caco‐2 cell viability was evaluated using the MTT assay after exposure to bacteria (MOI: 10), MSCs CM, MSCs CM–CS NPs, and CS NPs for 24 or 72 h. Following treatment, MTT solution was added for 3 h at 37°C, formazan crystals were dissolved in 100 μL DMSO, and absorbance was measured at 540 nm using an ELISA reader. All experiments were conducted in triplicate.

### 2.9. Statistical Analysis

All data were evaluated via GraphPad Prism software, Version 8 (GraphPad Software, Inc., 160 USA). The obtained findings were described using mean and standard deviation (SD). The significance level was considered at a *p* value of < 0.05. The difference among the different groups was examined using ANOVA analysis. Totally, the examinations were repeated three times.

## 3. Results

### 3.1. Characterization of MSCs CM and NPs

Based on the flow cytometric analysis findings, BM‐MSCs were negative for CD34 and CD45 but positive for CD44 and CD73 as MSC‐specific surface biomarkers. Size distribution and zeta potential of CS NPs were ∼ 85.2 nm and 32.1 mV, respectively (Figures 1(a) and 1(b)). SEM findings showed a near‐spherical shape with an irregular outer surface for MSCs CM–CS NPs as well as a spherical shape for CS NPs (Figures 2(a) and 2(b)).

FIGURE 1DLS analysis findings showed (a) a mean particle size of 85.2 nm and (b) a zeta potential of 32.1 mV for CS nanoparticles.

**Figure:**
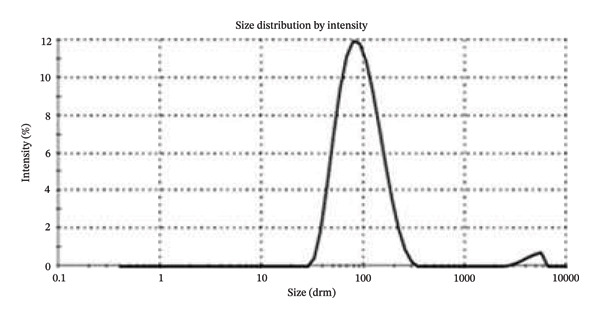
(a)

**Figure:**
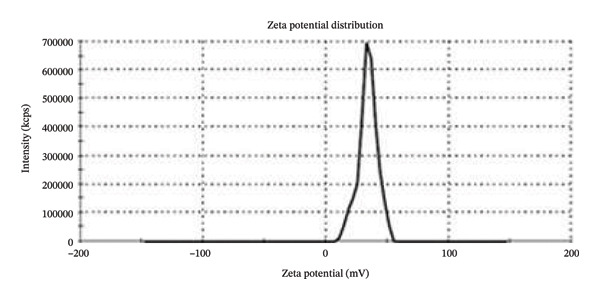
(b)

FIGURE 2SEM images showing the morphology of (a) the new composite (MSCs CM–CS NPs) and (b) CS nanoparticles.

**Figure:**
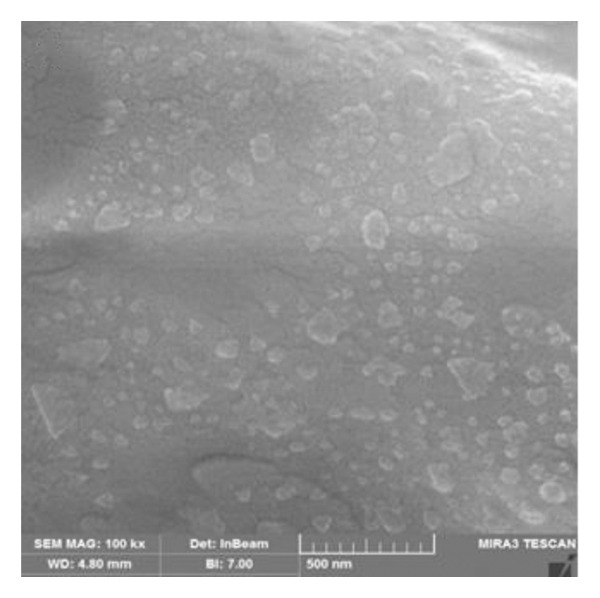
(a)

**Figure:**
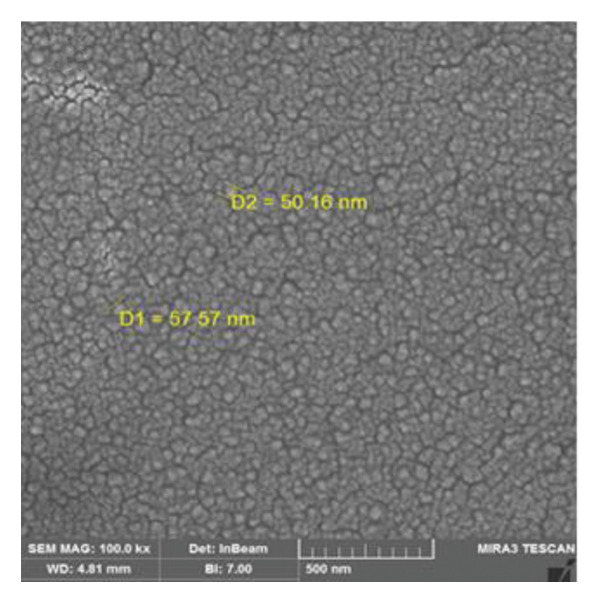
(b)

### 3.2. EE%

The composite (MSCs CM–CS NPs) had an EE of 71%, indicating the entrapment of large quantities of MSCs supernatant in CS NPs. The total released protein concentration was calculated from a standard curve.

### 3.3. In Vitro Release Assay

**FIGURE 3:**
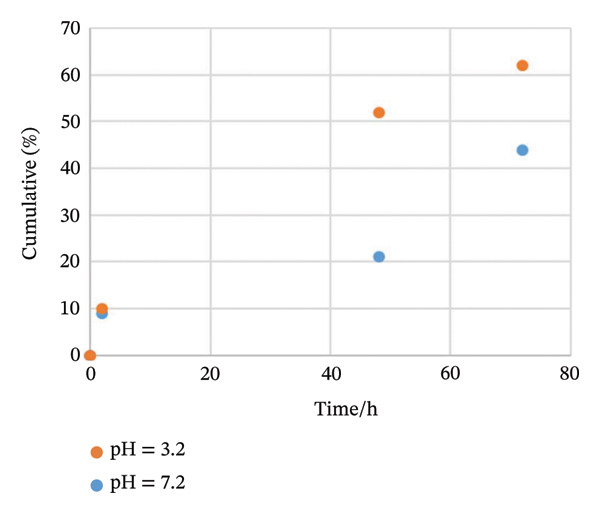
Release profile of MSCs CM from MSCs CM–CS NPs at pH 7.2 and 3.2 after 72 h by BCA assay kit in vitro.

The protein (MSCs CM) release profile from the new construct (MSCs CM–CS NPs) is presented in Figure 3. The amount of protein (MSCs CM) released from the new construct at pH 7.2 and 3.2 after 72 h was 62% and 44%, respectively. Therefore, as the pH decreased, the amount of protein released from the new construct also decreased (Figure 3).

### 3.4. Cell Viability of Caco‐2 Cells After Treatment With MDR Bacterial and Different Compounds

At an MOI of 10, about 80%–90% of Caco‐2 cells were infected, with viability remaining above 80% after 24 h. Treatment with MSCs CM, MSCs CM–CS NPs, and CS NPs showed similar results, with 24 h viabilities of 83%, 80%, and 77%, respectively. After 72 h, viability decreased to 65.18%, 61.46%, and 53.54%. Overall, MSCs CM maintained slightly higher cell viability than the other treatments.

### 3.5. Evaluation of Antimicrobial Potential by the Broth Microdilution Method

The antimicrobial activity of the new construct (MSCs CM–CS NPs) and its constituents (CS NPs and MSCs CM) against MDR bacterial pathogens (*P. aeruginosa, K. pneumoniae, E. cloacae*, MRSA, and *E. coli*) was investigated using the broth microdilution method.

The new construct (MSCs CM–CS NPs: 1000 μg/mL +0.05%) exhibited higher inhibitory activity than either MSCs CM (1000 μg/mL) or CS NPs (0.05%) alone; however, a statistically significant difference was observed only in comparison with MSCs CM at certain dilutions (*p* < 0.05) against bacterial growth (Figure 3).

**FIGURE 4:**
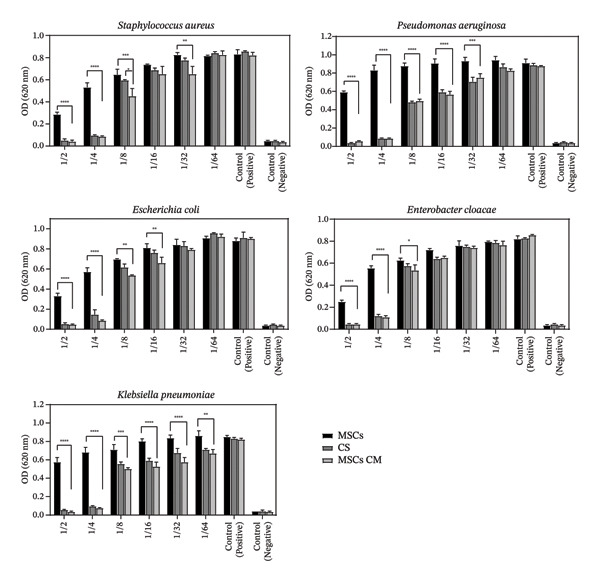
Antimicrobial potential of MSCs CM–CS NPs, MSCs supernatant, and CS nanoparticles against MDR bacteria at different dilutions (1:2–1:64). Values are represented as the average of three independent tests ± SD. ^∗^: *p* < 0.05, ^∗∗^: *p* < 0.01, ^∗∗∗^: *p* < 0.001, ^∗∗∗∗^: *p* < 0.0001, and ns: nonsignificant. MDR bacterial pathogens (*P.aeruginosa*, *K*. *pneumoniae*, *E*. *cloacae*, MRSA, and *E*. *coli* were used in this study. Significant differences were observed between MSCs and MSCs CM (between the first and last bands).

According to the results, the antibacterial activity of CS NPs and MSCs CM, each at specific concentrations, was lower than that of MSCs CM–CS NPs prepared with the same concentrations of their constituents. These results highlight the synergistic antimicrobial activity of the combination of MSCs supernatant and CS NPs. The antibacterial properties of the new construct and its components at different dilutions (1:2–1:64) against MDR pathogens are presented in Figure 4.

**FIGURE 5:**
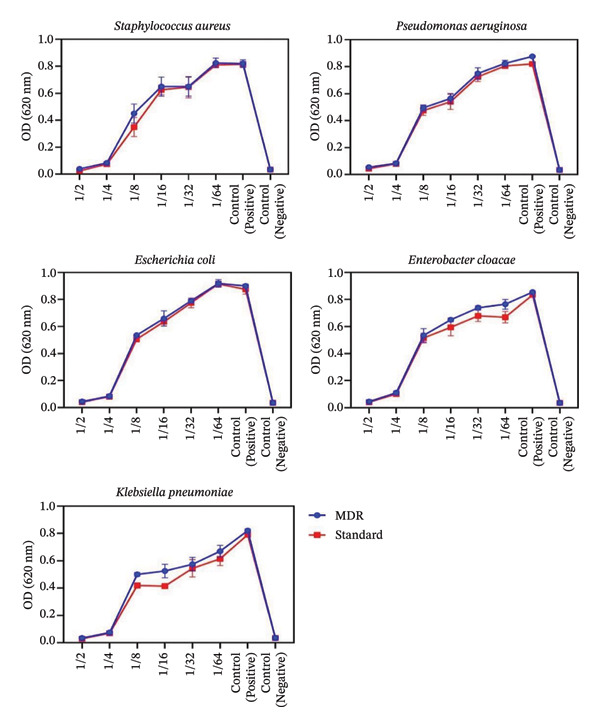
Antimicrobial potential of MSCs CM–CS NPs at different dilutions (1:2–1:64) against MDR and standard bacteria. Values are represented as the average of three independent tests ± SD. ^∗^: *p* < 0.05 (*P. aeruginosa*, *K*. *pneumoniae*, *E*. *cloacae*, MRSA, and *E*. *coli* were used in this study.

Figure 5 shows the antimicrobial activity of MSCs CM–CS NPs at different dilutions (1:2–1:64) against MDR and standard bacterial pathogens.

The MSCs CM–CS NPs supernatant showed significantly higher antibacterial activity than MSCs at the same tested dilutions, with the 1/2 dilution exhibiting the greatest effect (*p* < 0.05).

## 4. Discussion

MDR bacteria are the main public health concern worldwide, especially in developing countries. The current study results demonstrate that the use of composites (as versatile carriers) prepared using NPs and MSCs in the design of potential drugs against MDR bacteria, such as *P. aeruginosa*, *K. pneumoniae*, *E. cloacae*, *S. aureus*, and *E. coli,* may be an effective approach.

The prepared MSCs CM–CS NPs composite showed high antimicrobial activities against MDR bacteria (*P. aeruginosa*, *K. pneumoniae*, *E. cloacae*, *S. aureus*, and *E. coli*), which are regarded as a serious public health challenge. The antibacterial activity of the new construct (MSCs CM–CS NPs: 1000 μg/mL +0.05%) against MDR bacteria was significantly higher compared with MSCs CM (1000 μg/mL) and CS NPs (0.05%) each alone. The designed composite with targeted drug delivery exhibited high antibacterial activity, overcoming the critical weakness of other antimicrobials in reaching the target. It is estimated that if new antibiotics are not discovered, new and effective compounds will not be available in the future. In recent years, the need for alternative nonantibiotic treatment approaches and continuous antimicrobial surveillance to control infectious diseases has increased [16]. One of the promising novel strategies that could be used to kill and eliminate bacterial pathogens is cell therapy with mesenchymal stem cells (MSCs). As multipotent mature stem cells, MSCs have the potential for self‐renewal and differentiation into some mesenchymal lineages [17].

Recently, according to our study, multiple investigations have shown that human MSCs are capable of modulating innate immune cells and improving the outcomes of patients with severe infections. The mechanism of action of MSCs is through the secretion of various biologically active compounds with inflammatory, anti‐inflammatory, chemotactic, antimicrobial, and antiapoptotic properties. The innovative use of MSCs as a host‐isolated antibacterial agent against *E. coli* and *S. aureus* has been investigated in various studies. The primary mechanism of antimicrobial activity of MSCs is the secretion of LL‐37 as a cationic antimicrobial peptide [18, 19]. In another research carried out by Chow et al., the bactericidal potential of activated MSCs against MDR isolates was investigated. In their study, MSCs showed direct spontaneous antibacterial activities against MDR bacteria in vitro. Research has shown that MSCs could directly and indirectly eliminate chronic bacterial infections through immune‐mediated mechanisms [12]. Marx et al. demonstrated that equine MSCs increased the expression of AMPs (antimicrobial peptides) and CCL2. They showed that the antibacterial activity of equine keratinocytes increased by provoking the expression of antibacterial peptides in a skin biofilm explant model [20].

NPs could offer promising solutions to combat antimicrobial resistance and act as carriers for antibiotics and natural antimicrobial composites. In this study, the prepared CS NPs were characterized by SEM and DLS. Also, the zeta potential and size distribution of the synthesized CS NPs were confirmed using the DLS technique. The results of SEM and DLS analyses showed the spherical shape of CS NPs with a size distribution and zeta potential of ∼ 85.2 nm and 32.1 mV, respectively. SEM findings also showed a near‐spherical shape with an irregular outer surface for the new construct (MSCs CM–CS NPs).

Previous studies have described the antimicrobial potential of nanochitosan against some bacteria. The antibacterial activity of CS NPs has been investigated against *L. monocytogenes* and *Streptococcus* spp. [8, 9]. Studies have shown that CS NPs could disrupt the growth of different bacterial isolates [21–23]. CS NPs have a small size and natural bioactivity, and this property could make them distinctive.

Studies have revealed that the simultaneous use of MSCs with the most common antibiotic classes synergistically increases their antibacterial activities. However, few studies have been performed to investigate the therapeutic effect of MSCs along with other components on drug‐resistant chronic infections to inhibit bacterial growth [18].

In this study, low concentrations of CS NPs and MSCs supernatant were employed to synthesize the new composite (MSCs CM–CS NPs). SEM images and encapsulation efficiency of the composite were investigated. SEM images of the composite are shown in Figure 2(b). SEM findings showed a near‐spherical and irregular shape for the composite compared to CS NPs.

Using SEM analysis, the particle size of CS NPs was determined, while DLS analysis was used to assess both the particle size and zeta potential of CS NPs. The mean particle sizes obtained from SEM and DLS were 24.27 nm and 102 nm, respectively, with the SEM results being smaller than those from DLS. This size difference is likely due to the drying process in SEM, which may cause particle shrinkage, while DLS measures both the core and surface molecules of the NPs [24].

In one study, it was shown that CS NPs with an average size of 97 nm exhibited strong bactericidal activity against both Gram‐negative and Gram‐positive bacteria [25].

The composite (MSCs CM–CS NPs) had a significant EE, representing the entrapment of large quantities of MSCs supernatant in CS NPs. It was found that encapsulation efficiency was 71%. The rate of protein release from the composite was better in simulated intestinal conditions compared with the gastric acidic environment. The rate of protein release from MSCs CM–CS NPs decreased at acidic pH.

The amount of protein released from the composite during 72 h was higher at pH 7.2 compared with pH 3.2. Thus, the MSCs CM–CS construct is likely to have good stability in a gastric acidic environment, whereas the protein enclosed in MSCs CM–CS NPs is likely to be rapidly released in simulated intestinal conditions. Therefore, the limited protein release contributes to the long‐term release.

The MTT assay results at 24 and 72 h indicated that more than 80% of the cells remained viable, suggesting low cytotoxicity of the tested sample. These findings are consistent with previous studies by Schnur et al. [26] and Frigaard et al. [27], which also reported that similar components exhibited only mild cytotoxic effects on Caco‐2 cells.

The antimicrobial potential of the new construct (MSCs CM–CS NPs: 1000 μg/mL +0.05%) against five bacterial pathogens (*P.aeruginosa*, *K*.*pneumoniae*, *E*. *cloacae*, MRSA, and *E*. *coli* were used in this study) was evaluated using the broth microdilution method. The antimicrobial activity of MSCs CM–CS NPs was significant at dilutions of 1:2–1:81:32 against methicillin‐resistant *S. aureus* (MRSA), at dilutions of 1:2–1:32 against *P*. *aeruginosa*, and at dilutions of 1:2–1:16 and 1:2–1:8 against *E. coli* and *Enterobacter*, respectively. The most significant antimicrobial activity of MSCs CM–CS NPs was against *Klebsiella* at dilutions of 1:2–1:64. The antibacterial activity of MSCs CM–CS NPs at dilutions of 1:2–1:64 against five bacterial pathogens was higher than that of CS NPs.

Using the broth microdilution method, Bahroudi et al. showed that unstimulated MSCs supernatant exhibited remarkable dose‐dependent antibacterial activities (1:8–1:128) against *V. cholerae*. They proved that the bacterial load in the bacterial suspension was reduced from 10^8^ to 10^7^ CFU/mL [10].

The antimicrobial activity shows that the sample was more effective against standard strains than MDR strains, likely due to resistance mechanisms in MDR bacteria. Also, the results show that the prepared MSCs CM–CS NPs composite at higher concentrations has potential antibacterial activity against MDR bacteria and, therefore, could be used as an adjunctive antimicrobial agent to treat MDR infections. The potent antibacterial activity of the new construct against MDR bacteria could be attributed to the synergistic effect of its two constituents. Several studies have demonstrated that MSC‐containing formulations, such as chitosan–alginate membranes incorporating DHA, significantly improve antibiofilm and antibacterial activities against *Pseudomonas aeruginosa*, enhance MSC viability, accelerate burn wound healing, and reduce inflammation [28].

Moreover, the combination of MSCs with Ophiophagus hannah L‐amino acid oxidase (Oh‐LAAO) showed a significant reduction in methicillin‐resistant *Staphylococcus aureus* (MRSA) bacterial load and accelerated wound healing [29].

Other research has highlighted that MSCs combined with major antibiotic classes, including aminoglycosides, cephalosporins, vancomycin, fluoroquinolones, and chloramphenicol, substantially enhance bactericidal activity against *Staphylococcus* species and *Escherichia coli* [18].

Additionally, antibiotic‐loaded hydrogels containing MSCs and minocycline have been shown to reduce the production of the antimicrobial peptide LL‐37, boost antimicrobial effects, increase IL‐6 production and bacterial internalization, decrease *Staphylococcus aureus* bioburden in inoculated wounds, and promote re‐epithelialization [30].

Finally, CMs from MSCs combined with CS NPs exhibited more efficient inhibition of biofilm formation by *V. cholerae*, improved antimicrobial activity, and demonstrated optimal release profiles within the intestinal lumen [11].

The findings of various studies regarding the antimicrobial activities of MSCs against bacterial pathogens are different. The difference in the findings of various studies could be attributed to the difference in the concentration of MSCs and the type of bacterial isolates used. Another important factor influencing the antibacterial potential of composites may be the type of materials integrated with MSCs in the composite.

The results of this study have significant implications for the control of MDR infections, which are progressively increasing in terms of incidence and severity. Several studies have been conducted to assess the efficacy of MSCs in the treatment of acute infections; however, the enhancement mechanisms are still not well understood [31, 32].

In fact, various complementary mechanisms are probably involved in the antimicrobial activities of MSCs against MDR bacteria, including the secretion of local antimicrobial peptides (direct effect) and systemic stimulation of host innate immunity (indirect effect) [18]. The present study findings highlight the potential of MSCs CM–CS NPs as a growth inhibitor against MDR bacteria.

In short, this study results show that the new construct (MSCs CM–CS NPs: 1000 μg/mL +0.05%) could be used as an antibacterial agent against MDR clinical isolates. The primary limitation of the current research was the lack of an in vivo study, which is proposed to be done in future research.

## 5. Conclusion

The prepared MSCs CM–CS NPs composite exhibited strong antimicrobial activity against MDR bacteria, including *P*. *aeruginosa*, *K. pneumoniae*, *E. cloacae*, *S. aureus*, and *E. coli,* a major public health concern, especially in developing countries. SEM and DLS analyses showed that CS NPs had a spherical shape with an average size of ∼85.2 nm and a zeta potential of 32.1 mV. The composite structure appeared near‐spherical with an irregular surface. The MSCs CM–CS NPs showed high EE and better protein release under simulated intestinal conditions than in acidic environments. Antibacterial activity of the composite (MSCs CM–CS NPs: 1000 μg/mL + 0.05%) was significantly higher than either MSCs CM or CS NPs alone. The targeted delivery system improved efficacy, suggesting a synergistic effect. Importantly, due to its nonantibiotic nature, the composite may avoid traditional resistance mechanisms. Therefore, it shows promise as a novel therapeutic or adjunctive antibacterial agent. However, further in vivo studies are needed to explore its antibacterial mechanisms and clinical applications.

**Nomenclature**
WHO: World Health Organization
MDR: Multidrug‐resistance
DLS: Dynamic light scattering
SEM: Scanning electron microscopy
CFU: Colony‐forming units
CS NPs: Chitosan nanoparticles
CM: Conditioned medium
MSCs: Mesenchymal stem cells
MSCs CM: Mesenchymal stem cell‐derived conditioned medium
PBS: Phosphate‐buffered saline
EMB: Eosin methylene blue
SS agar: *Salmonella*–*Shigella* agar
EE: Entrapment efficiency
IHC: Immunohistochemistry
BHI: Brain–heart infusion
LB: Luria–Bertani

## Funding

The present research was supported by Kashan University of Medical Sciences, Kashan (IR.KAUMS.MEDNT.REC.1403.159).

## Disclosure

All authors have reviewed and approved the final version of the article.

## Ethics Statement

The current research was approved by the Ethics Committee of Kashan University of Medical Sciences.

## Consent

The authors have nothing to report.

## Conflicts of Interest

The authors declare no conflicts of interest.

## Data Availability

The data that support the findings of this study are available from the corresponding author upon reasonable request.

## References

[1] Vila J. , Multidrug-Resistant Bacteria Without Borders: Role of International Trips in the Spread of Multidrug-Resistant Bacteria, Journal of Travel Medicine. (2015) 22, no. 5, 289–291, 10.1111/jtm.12231, 2-s2.0-84941082042. 26333539

[2] Rotello V. M. , Nanomaterials for Fighting Multidrug-Resistant Biofilm Infections, BME Frontiers. (2023) 4, 10.34133/bmef.0017. PMC10521699 37849666

[3] Yoneyama H. and Katsumata R. , Antibiotic Resistance in Bacteria and Its Future for Novel Antibiotic Development, Bioscience Biotechnology and Biochemistry. (2006) 70, no. 5, 1060–1075, 10.1271/bbb.70.1060, 2-s2.0-33646818699. 16717405

[4] Jabir M. S. , Abood N. A. , Jawad M. H. et al., Gold Nanoparticles Loaded TNF-α and CALNN Peptide as a Drug Delivery System and Promising Therapeutic Agent for Breast Cancer Cells, Materials Technology. (2022) 37, no. 14, 3152–3166, 10.1080/10667857.2022.2133073.

[5] Mahdi L. H. , Hasoon B. A. , Sulaiman G. M. et al., Anti-Microbial Efficacy of l-Glutaminase (EC 3.5. 1.2) Against Multidrug-Resistant *Pseudomonas aeruginosa* Infection, Journal of Antibiotics. (2024) 77, no. 2, 111–119, 10.1038/s41429-023-00678-z. 38017084

[6] Derakhshan-Sefidi M. , Bakhshi B. , and Rasekhi A. , Thiolated Chitosan Nanoparticles Encapsulated Nisin and Selenium: Antimicrobial/Antibiofilm/Anti-Attachment/Immunomodulatory Multi-Functional Agent, BioMed Central Microbiology. (2024) 24, no. 1, 10.1186/s12866-024-03400-7. PMC11241873 38997643

[7] Kahdim Q. S. , Abdelmoula N. , Al-Karagoly H. , Albukhaty S. , and Al-Saaidi J. , Fabrication of a Polycaprolactone/Chitosan Nanofibrous Scaffold Loaded With Nigella Sativa Extract for Biomedical Applications, BioTechnologia. (2023) 12, no. 1, 10.3390/biotech12010019. PMC9944449 36810446

[8] Rad S. K. , Assmar M. , Mirpour M. , and Razavi M. R. , Evaluation of Chitosan Nanoparticle Antimicrobial Effect on Isolated Listeria Monocytogenes Bacteria From Pregnant Women and L. Monocytogenes ATCC 7644, Iranian Journal of Public Health. (2022) 51, no. 12, 10.18502/ijph.v51i12.11469. PMC9874196 36742246

[9] Aliasghari A. , Khorasgani M. R. , Vaezifar S. , Rahimi F. , Younesi H. , and Khoroushi M. , Evaluation of Antibacterial Efficiency of Chitosan and Chitosan Nanoparticles on Cariogenic Streptococci: An in Vitro Study, Iranian Journal of Microbiology. (2016) 8, no. 2, 93–100. 27307974 PMC4906725

[10] Bahroudi M. , Bakhshi B. , Soudi S. , and Najar-Peerayeh S. , Antibacterial and Antibiofilm Activity of Bone Marrow-Derived Human Mesenchymal Stem Cells Secretome Against *Vibrio cholerae* , Microbial Pathogenesis. (2020) 139, 10.1016/j.micpath.2019.103867. 31712121

[11] Saberpour M. , Bakhshi B. , and Najar-Peerayeh S. , Evaluation of the Antimicrobial and Antibiofilm Effect of Chitosan Nanoparticles as Carrier for Supernatant of Mesenchymal Stem Cells on Multidrug-Resistant *Vibrio cholerae* , Infection and Drug Resistance. (2020) 13, 2251–2260, 10.2147/IDR.S244990. 32765001 PMC7367937

[12] Chow L. , Johnson V. , Impastato R. , Coy J. , Strumpf A. , and Dow S. , Antibacterial Activity of Human Mesenchymal Stem Cells Mediated Directly by Constitutively Secreted Factors and Indirectly by Activation of Innate Immune Effector Cells, Stem Cells Translational Medicine. (2020) 9, no. 2, 235–249, 10.1002/sctm.19-0092. 31702119 PMC6988770

[13] Tawre M. S. , Shiledar A. , Satpute S. K. , Ahire K. , Ghosh S. , and Pardesi K. , Synergistic and Antibiofilm Potential of Curcuma aromatica Derived Silver Nanoparticles in Combination With Antibiotics Against Multidrug-Resistant Pathogens, Frontiers in Chemistry. (2022) 10, 10.3389/fchem.2022.1029056. PMC9682076 36438875

[14] Piras A. M. , Maisetta G. , Sandreschi S. et al., Chitosan Nanoparticles Loaded With the Antimicrobial Peptide Temporin B Exert a Long-Term Antibacterial Activity in Vitro Against Clinical Isolates of Staphylococcus Epidermidis, Frontiers in Microbiology. (2015) 6, 10.3389/fmicb.2015.00372, 2-s2.0-84930941872. PMC4412066 25972852

[15] Rabima R. and Sari M. P. , Entrapment Efficiency and Drug Loading of Curcumin Nanostructured Lipid Carrier (NLC) Formula, Pharmacia. (2019) 9, no. 2, 299–306, 10.12928/pharmaciana.v9i2.13070.

[16] Thabit A. K. , Crandon J. L. , and Nicolau D. P. , Antimicrobial Resistance: Impact on Clinical and Economic Outcomes and the Need for New Antimicrobials, Expert Opinion on Pharmacotherapy. (2015) 16, no. 2, 159–177, 10.1517/14656566.2015.993381, 2-s2.0-84921051811. 25496207

[17] Mundra V. , Gerling I. C. , and Mahato R. I. , Mesenchymal Stem Cell-Based Therapy, Molecular Pharmaceutics. (2013) 10, no. 1, 77–89, 10.1021/mp3005148, 2-s2.0-84872139117. 23215004 PMC3549356

[18] Johnson V. , Chow L. , Harrison J. , Soontararak S. , and Dow S. , Activated Mesenchymal Stromal Cell Therapy for Treatment of Multi-Drug Resistant Bacterial Infections in Dogs, Frontiers in Veterinary Science. (2022) 9, 10.3389/fvets.2022.925701. PMC9260693 35812842

[19] Yagi H. , Chen A. F. , Hirsch D. et al., Antimicrobial Activity of Mesenchymal Stem Cells Against *Staphylococcus aureus* , Stem Cell Research & Therapy. (2020) 11, no. 1, 1–12, 10.1186/s13287-020-01807-3. 32680544 PMC7367313

[20] Marx C. , Gardner S. , Harman R. M. , Wagner B. , and Van de Walle G. R. , Mesenchymal Stromal Cell-Secreted CCL2 Promotes Antibacterial Defense Mechanisms Through Increased Antimicrobial Peptide Expression in Keratinocytes, Stem Cells Translational Medicine. (2021) 10, no. 12, 1666–1679, 10.1002/sctm.21-0058. 34528765 PMC8641085

[21] Alqahtani F. , Aleanizy F. , El Tahir E. et al., Antibacterial Activity of Chitosan Nanoparticles Against Pathogenic N. Gonorrhoea, International Journal of Nanomedicine. (2020) 15, 7877–7887, 10.2147/IJN.S272736. 33116506 PMC7568623

[22] Ikono R. , Vibriani A. , Wibowo I. et al., Nanochitosan Antimicrobial Activity Against Streptococcus Mutans and Candida Albicans Dual-Species Biofilms, BioMed Central microbiology Research Notes. (2019) 12, no. 1, 1–7, 10.1186/s13104-019-4422-x, 2-s2.0-85068918816. PMC6613267 31287001

[23] Al-Zahrani S. S. , Bora R. S. , and Al-Garni S. M. , Antimicrobial Activity of Chitosan Nanoparticles, Biotechnology & Biotechnological Equipment. (2021) 35, no. 1, 1874–1880, 10.1080/13102818.2022.2027816.

[24] Rema S. S. , Vadavanath Prabhakaran V. , Premachandran Latha R. , and Kozhiparambil Gopalan R. , Preparation and Characterization of Selenium Incorporated Guar Gum Nanoparticle and Its Interaction with H9c2 Cells, Public Library of Science One. (2013) 8, 10.1371/journal.pone.0074411, 2-s2.0-84884759765. PMC3787042 24098647

[25] Nasr M. , Diab A. , Roshdy N. , and Hussein A. , Assessment of Antimicrobial Efficacy of Nano Chitosan, Chlorhexidine, Chlorhexidine/Nano Chitosan Combination Versus Sodium Hypochlorite Irrigation in Patients With Necrotic Mandibular Premolars: A Randomized Clinical Trial, Open Access Macedonian Journal of Medical Sciences. (2021) 9, 235–242, 10.3889/oamjms.2021.7070.

[26] Schnur S. , Hans F. , Dehne A. et al., The Potential of Epigallocatechin-3-Gallate (EGCG) as Complementary Medicine for the Treatment of Inflammatory Bowel Disease, Pharmaceuticals. (2023) 16, no. 5, 10.3390/ph16050748. PMC10224516 37242530

[27] Frigaard J. , Jensen J. L. , Galtung H. K. , and Hiorth M. , The Potential of Chitosan in Nanomedicine: An Overview of the Cytotoxicity of Chitosan Based Nanoparticles, Frontiers in Pharmacology. (2022) 13, 10.3389/fphar.2022.880377. PMC9115560 35600854

[28] Ghaneialvar H. , Kayumov A. , Aboualigalehdari E. et al., Docosahexaenoic Acid-Loaded Chitosan/Alginate Membrane Reduces Biofilm Formation by *P. aeruginosa* and Promotes MSC-Mediated Burn Wound Healing, Journal of Biomaterials Applications. (2023) 37, no. 8, 1458–1469, 10.1177/08853282221131130. 36189675

[29] Mot Y. Y. , Othman I. , and Sharifah S. H. , Synergistic Antibacterial Effect of Co-Administering Adipose-Derived Mesenchymal Stromal Cells and Ophiophagus Hannah L-Amino Acid Oxidase in a Mouse Model of Methicillin-Resistant Staphylococcus Aureus-Infected Wounds, Stem Cell Research & Therapy. (2017) 8, no. 1, 10.1186/s13287-016-0457-2, 2-s2.0-85010931939. PMC5259957 28114965

[30] Guerra A. D. , Rose W. E. , Hematti P. , and Kao W. J. , Minocycline Modulates NFκB Phosphorylation and Enhances Antimicrobial Activity Against *Staphylococcus aureus* in Mesenchymal Stromal/Stem Cells, Stem Cell Research & Therapy. (2017) 8, no. 1, 10.1186/s13287-017-0623-1, 2-s2.0-85025160429. PMC5521110 28732530

[31] McIntyre L. A. , Stewart D. J. , Mei S. H. et al., Cellular Immunotherapy for Septic Shock. A Phase I Clinical Trial, American Journal of Respiratory and Critical Care Medicine. (2018) 197, no. 3, 337–347, 10.1164/rccm.201705-1006OC, 2-s2.0-85045273837. 28960096

[32] Fernández-Francos S. , Eiro N. , González-Galiano N. , and Vizoso F. J. , Mesenchymal Stem Cell-Based Therapy as an Alternative to the Treatment of Acute Respiratory Distress Syndrome: Current Evidence and Future Perspectives, International Journal of Molecular Sciences. (2021) 22, no. 15, 10.3390/ijms22157850. PMC8346146 34360616

